# Daily physical activity predicts degree of insulin resistance: a cross-sectional observational study using the 2003–2004 National Health and Nutrition Examination Survey

**DOI:** 10.1186/1479-5868-10-10

**Published:** 2013-01-28

**Authors:** Rachael K Nelson, Jeffrey F Horowitz, Robert G Holleman, Ann M Swartz, Scott J Strath, Andrea M Kriska, Caroline R Richardson

**Affiliations:** 1 School of Kinesiology, University of Michigan, Ann Arbor, MI USA; 2 VA Center for Clinical Management Research, VA HSR&D Center of Excellence, HSR&D/SMITREC (152), 2215 Fuller Rd, P.O. Box 130170, Ann Arbor, MI 48113-0170, USA; 3 Department of Human Movement Sciences, University of Wisconsin-Milwaukee, Milwaukee, WI, 53201-0413, USA; 4 Department of Epidemiology, Graduate School of Public Health, 505 B, University of Pittsburg, 130 DeSoto Street, Pittsburg, PA, 15261, USA; 5 Department of Family Medicine, University of Michigan Medical School, 1018 Fuller St, Ann Arbor, MI 48104-1213, USA

**Keywords:** Ambulatory monitoring, Physical fitness, Glucose tolerance test, Adiposity

## Abstract

**Background:**

This study examined the independent association of objectively measured physical activity on insulin resistance while controlling for confounding variables including: cardiorespiratory fitness, adiposity, sex, age, and smoking status.

**Methods:**

Data were obtained from National Health and Nutrition Examination Survey 2003–2004, a cross-sectional observational study conducted by the National Center for Health Statistics of the Centers for Disease Control that uses a stratified, multistage probability design to obtain a nationally representative sample of the U.S. population. The analysis included 402 healthy U.S. adults with valid accelerometer, cardiorespiratory fitness, and fasting plasma glucose and insulin concentrations. After controlling for relevant confounding variables we performed a multiple linear regression to predict homeostatic model of insulin resistance (HOMA-IR) based on average daily minutes of moderate-to-vigorous physical activity (MVPA).

**Results:**

In our bivariate models, MVPA, cardiorespiratory fitness and body fat percentage were all significantly correlated with log HOMA-IR. In the complete model including MVPA and relevant confounding variables, there were strong and significant associations between MVPA and log HOMA-IR (β= −0.1607, P=0.004). In contrast the association between cardiorespiratory fitness and log HOMA-IR was not significant.

**Conclusion:**

When using an objective measure of physical activity the amount of time engaged in daily physical activity was associated with lower insulin resistance, whereas higher cardiorespiratory fitness was not. These results suggest that the amount of time engaged in physical activity may be an important determinant for improving glucose metabolism.

## Background

The incidence of obesity-related diseases, such as type 2 diabetes is increasing in parallel with the alarming rise in the prevalence of obesity [1]. Lifestyle programs involving weight loss and exercise are often found to improve insulin resistance (IR) in individuals with diabetes, and also to prevent or delay the onset of diabetes in those at risk of developing the disease [2]. Although weight loss can markedly improve IR, exercise can also improve IR even in the absence of weight loss. Additionally, although improved cardiovascular “fitness” in overweight and obese individuals is linked with a reduced incidence of diabetes, exercise can also improve IR without enhancing cardiovascular fitness [3]. For example, a single session of exercise can have a profound improvement on IR that can persist for several hours and even days [4,5]. Collectively this evidence suggests that exercise, per se, provides a potent stimulus for improving IR. Therefore, promoting a lifestyle change to increase regular physical activity (PA), even if it is not sufficient to induce weight loss or improve cardiovascular fitness, may be a viable and realistic intervention option aimed at reducing diabetes risk.

“PA” is broadly defined to include any bodily movement produced by skeletal muscle that increases energy expenditure [6]. Until recently it has been difficult to quantify ambulatory PA in free-living individuals. Large cohort studies have traditionally used self-reported surveys to measure PA, contributing to errors in determining the intensity and duration of PA [7,8]. However, objectively measured PA has been shown to be a better predictor of metabolic health than self-reported PA [9]. Accelerometers, currently the gold standard for objectively measuring PA in a free-living population [10], were incorporated into the National Health and Nutrition Examination Survey (NHANES) 2003–2004 survey, a nationally representative sample of residence in the U.S. Analysis of fasting blood samples (including plasma concentrations of insulin and glucose), and other variables known to influence IR (i.e. cardiorespiratory fitness, and total body adiposity) were also determined for a subset of healthy participants in this NHANES cohort. The objective measurement of these constructs in a large representative cohort of U.S. adults allows more precise estimates than in previous studies along with an opportunity to access lower intensity PA that is difficult to quantify by self-report. Therefore, the aim of our study was to examine the independent association of objectively measured PA on IR while controlling for potential confounding variables in a representative sample of healthy U.S. adults.

## Methods

### Inclusion criteria

**Figure 1:**
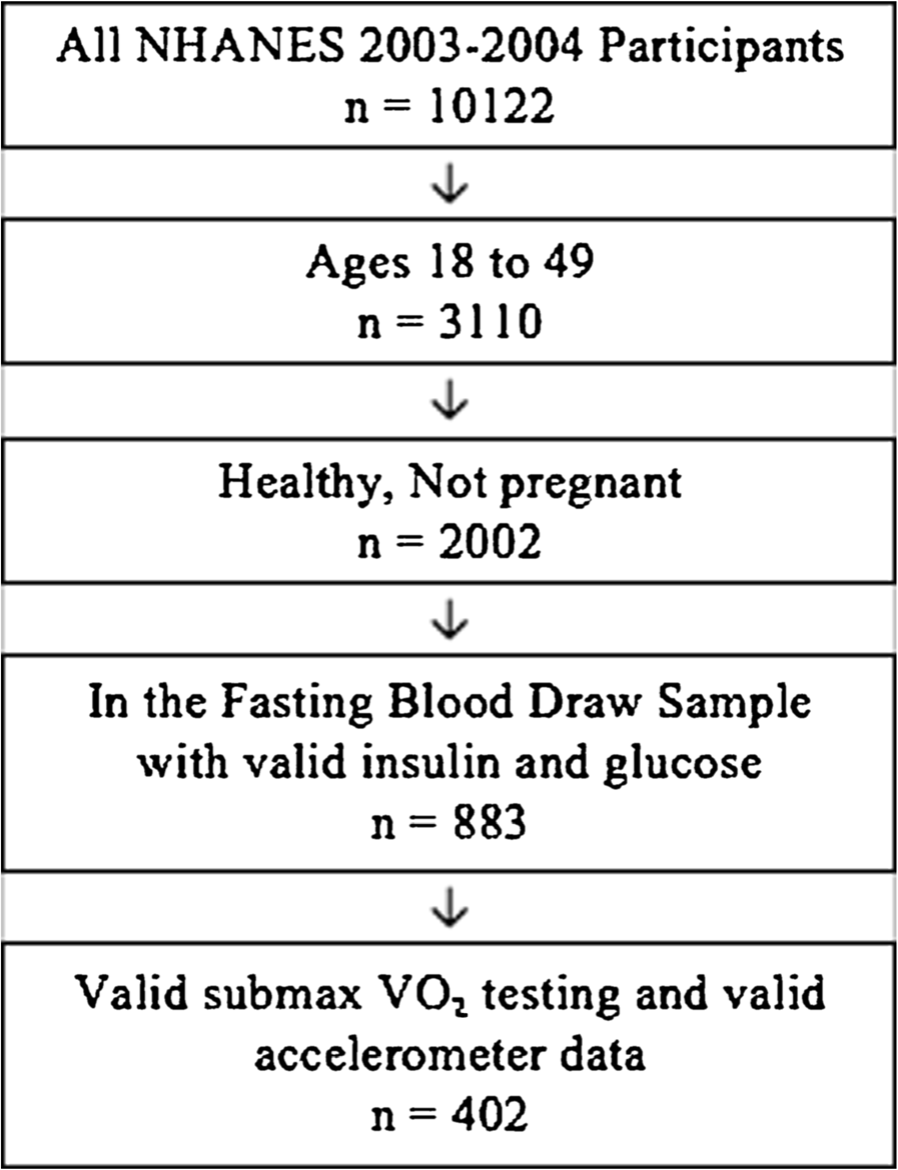
Inclusion (subsample) flow chart.

NHANES is a cross-sectional observational study conducted by the National Center for Health Statistics of the Centers for Disease Control and Prevention that uses a stratified, multistage probability design to obtain a nationally representative sample of the U.S. population. The survey population included randomly selected households within clusters of neighborhoods. Of the 12,761 individuals selected during the 2003–2004 survey, 10,122 individuals agreed to participate. The NHANES 2003–2004 survey included an interview, physical examination and laboratory testing conducted by trained staff. Only non-pregnant adults aged 18–49 without a history of diabetes (or taking medication to treat diabetes), cardiovascular or renal disease, stroke or emphysema were included in this analysis. Because participants were scheduled for either a morning blood draw after a 9-hour fast or an afternoon blood draw after a 6-hour fast, a fasting questionnaire was used to determine whether or not participants met the fasting requirements. Therefore, fasting glucose and insulin used to calculate the primary outcome of homeostatic model of IR (HOMA-IR) were only available on approximately half of the NHANES participants who were classified as fasted. Also, only participants who successfully completed submaximal VO2 fitness test in order to determine cardiorespiratory fitness were included in this analysis (Figure 1).

### Assessment of insulin resistance

Blood samples were obtained by trained medical personal in mobile examination centers after a 6- or 9-hour fast. Samples were centrifuged; the plasma from each sample was placed into storage test tubes, shipped to the University of Missouri-Columbia (Columbia, Missouri), and stored at −70° until analysis. Plasma glucose concentration was determined by hexokinase enzyme method. Plasma insulin concentrations were measured with Tosoh AIA-PACK IRI (Toyama, Japan) two-site immunoenzymometric assay. Individuals with fasting insulin concentrations < 2 μIU/mL or > 100 μIU/mL (n = 34) were considered outliers and were excluded from the analysis. HOMA-IR (the product of fasting plasma glucose and insulin concentrations divided by 22.5 [mM*μIU/mL/22.5]) was used as a composite index for IR [11].

### Physical activity

PA was monitored by an Actigraph AM-7164 accelerometer (formerly the CSA/MTI AM-7164, manufactured by ActiGraph of Ft. Walton Beach, FL), which is a pager-sized device powered by a small lithium battery and attached to an elasticized belt worn on the right hip [12]. The accelerometer measured the duration and intensity of PA by capturing the magnitude of acceleration (intensity) and summing the magnitudes (intensity counts) within a specified time interval (epoch). A one-minute epoch was used by NHANES [13]. Participants were asked to wear the device for seven consecutive days while they were awake and to remove it while swimming or bathing. Monitors were returned by express mail to NHANES, where data were downloaded and the device was checked to determine whether it was still within manufacturer’s calibration specifications. NHANES used standardized data quality procedures to assess validity and reliability of Actigraph ACC data [13]. Our analysis included PA data from participants who wore the accelerometer for at least 600 minutes on four or more days of the week. Any block of time greater than or equal to 60 minutes where the activity count was equal to zero was considered time when the monitor was not worn. Each minute of accelerometer data was coded based on the recorded activity counts for that minute. Minutes with ≥1952 activity counts were coded as moderate-to-vigorous intensity PA (MVPA) and ≥260 and <1952 activity counts were coded as light PA [14]. We summed the number of minutes at these intensities over the entire day. This activity did not have to represent clearly defined sessions of exercise or be sequential and were thus accumulated throughout the day. Minutes of activity were divided by 30 to reduce the risk of rounding error in regression betas due to the fine scale of activity minutes. This calculation yields a measure of PA in which each unit represents approximately 30 minutes of PA per day. Changes to the units do not affect the associations described by our analysis.

### Cardiorespiratory fitness

A multiple-stage submaximal treadmill exercise test was employed to estimate maximal oxygen consumption (VO2max), which was used as the marker for cardiorespiratory fitness. Per NHANES protocol, inclusion criteria for exercise testing were based on medical conditions, medication and physical limitations determined during household interviews, and limited to adults 18–49 years who did not have any known cardiac conditions. A detailed list of inclusion criteria and submaximal exercise testing procedures can be found elsewhere [15]. Briefly, the exercise test included a two-minute warm-up on the treadmill, two separate three-minute submaximal exercise stages and a two-minute cool-down. Heart rate associated with known workloads at the end of each stage were used to estimate VO2max, as previously described [15]. Grade and speed for each stage were selected based on age, sex, body mass, and self reported PA. Heart rate was monitored throughout the test by four electrodes attached to the participant’s thorax and abdomen. Individuals with non-physiological estimates of VO2 max (> 100 ml/kg/min, n=3) were excluded from the analysis.

### Anthropometric measures

A wall-mounted stadiometer and a digital floor scale were used to measure height and weight, respectively, and calibrated as previously described [16]. Body fat percentage was determined by dual-energy X-ray absorptiometry (DEXA) using Hologic QDR-4500A fan-beam densitometer (Hologic, Inc., Bedford, Massachusetts) [17]. The DEXA was performed by a certified radiology technologist, and the densitometer was calibrated daily with a Hologic Anthropomorphic Spine Phantom as directed by the manufacturer.

### Smoking status

Smoking status was determined by measuring plasma serum levels of cotinine, which is a major metabolite of nicotine. Individuals with cotinine levels > 10 ng/ml were coded as being current smokers.

### Participant characteristics

Age at the time of the survey was calculated from the participant’s self-reported date of birth. Participants were classified into 1 of 4 categories for race/ethnicity based on self-reported background: Non-Hispanic white, Non-Hispanic black, Mexican American, or other.

### Statistical analysis

Descriptive statistics include means, standard deviations, and range for continuous variables (glucose, insulin, HOMA-IR, PA, VO2max, percent body fat, and age). Frequencies were calculated for categorical variables (i.e. sex and race/ethnicity). Because HOMA-IR was not normally distributed, we used log HOMA-IR as the primary outcome variable in our regressions.

Multiple linear regressions were performed with log HOMA-IR as the dependent variable. HOMA-IR was transformed using the log function to correct for the skewed distribution. Minutes of MVPA and VO2max were the primary predictor variables. Appropriate confounding variables including: adiposity, sex, and age were also included in our complete model. Model parameters were also stratified by sex. Race/ethnicity and smoking status did not significantly impact model estimates and was not included in any reported models. Adjusted r-squared values were calculated during the addition of confounding variables to our complete model. We also examined variance inflation factors to determine multicollinearity in our complete model.

Predicted mean values of HOMA-IR with confidence intervals were calculated for various levels of PA using the complete multiple linear regression including PA, cardiorespiratory fitness, percent body fat, age, and sex as independent variables.

All analysis took into consideration NHANES complex survey design including: weighting, stratification, and clustering. Sample weights for the NHANES 2003–2004 fasting sample were used. For all analysis, significance was set at <0.05. Statistical analysis was performed using Stata 11.0 for Windows (StataCorp LP, College Station, TX, 2006).

## Results

### Participant characteristics

Of the 10,122 participants in the 2003–2004 NHANES sample, 883 were healthy, non-pregnant adults between the ages of 18–49 who were scheduled for a morning fasting blood draw. Of those 883 participants, 402 had valid data for HOMA-IR calculations, estimated VO2 max, and PA (Figure 1). Categorical and continuous summary statistics of the final sample are presented in Table 1. The 481 individuals who were excluded from the analysis were slightly older, heavier and more likely to be non-white than the 402 with complete data. (Table 1).

**Table 1:** Participant demographics, anthropometric, metabolic, physical activity characteristics and cardiorespiratory fitness (n=402)

**Variable**	**Final Cohort**	**Range**	**Excluded Individuals**
	**Mean ± Linearized SE (n=402)**	**(n=402)**	**Mean ± Linearized SE (n=481)**
Sex (%)			
Male	53.0		49.0
Female	47.0		51.0
Race (%)			
Non-Hispanic white	74.0		68.0*
Non-Hispanic black	10.0		14.0*
Mexican American	10.0		8.0*
Other	7.0		10.0*
Smoking Status			
Smokers	29.0		37.0
Non-smokers	71.0		63.0
Age (yrs.)	32.6 ± 0.56	18–49	34.0 ± 0.51*
Mass (kg)	79.7 ± 1.16	42.8–156.7	82.8 ± 0.95
BMI^a^ (kg/m2)	26.9 ± 0.26	16.0–50.1	28.3 ± 0.29*
Body Fat (%)	31.2 ± 0.40	12.2–51.8	33.2 ± 0.42*
Fasting glucose (mmol/L)	5.1 ± 0.03	3.8–12.1	
Fasting insulin (μU/mL)	9.4 ± 0.49	2.0–61.2	
HOMA–IR^b^	2.1 ± 0.11	0.2–17.3	
Average MVPA^c ^(min)	32.1 ± 1.23	0.0–134.0	
Estimated VO2max^d ^(ml/kg/min)	39.7 ± 0.54	21.4–78.9	

^a ^body mass index.

^b ^homeostatic model assessment of insulin resistance.

^c ^moderate-to-vigorous physical activity.

^d ^maximal oxygen consumption.

*Statistically significant difference between final cohort (n=402) with valid measures of HOMA-IR, cardiorespiratory fitness, and physical activity and those excluded due to missing or invalid data.

### Regression models

**Figure 2:**
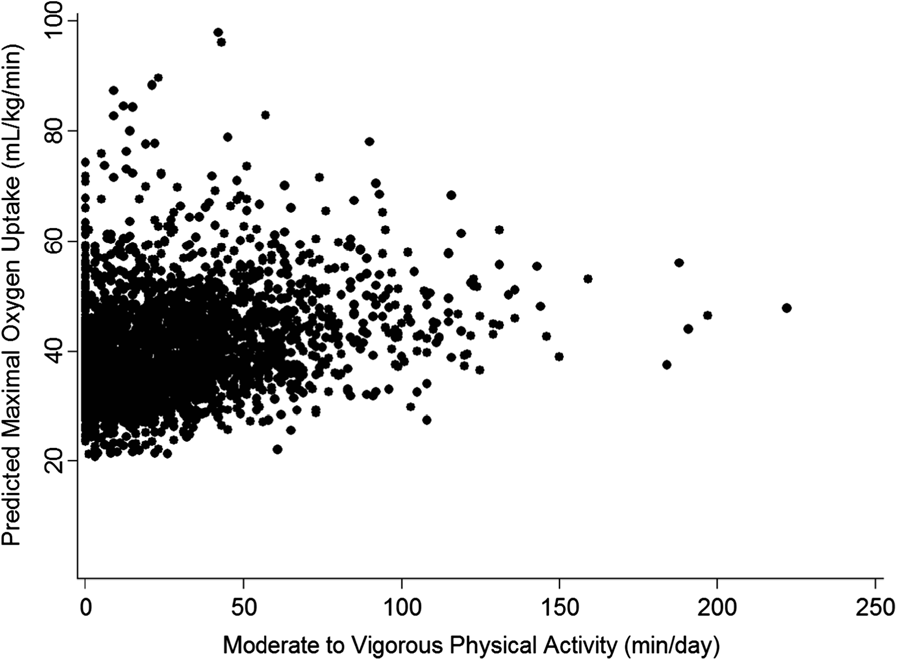
Relationship between average minutes of daily MVPA and cardiorespiratory fitness.

Parameter estimates for the four different regression models were presented in Table 2. We did observe a weak but significant relationship between cardiorespiratory fitness and physical activity (r2=0.1065, P<0.01; Figure 2). Importantly variables in our complete model did not demonstrate multicollinearity with an average variance inflation factor of 1.55 (range: 1.05–2.19). In bivariate models, MVPA and cardiorespiratory fitness were all significantly correlated with log HOMA-IR with higher levels of MVPA (β=−0.224, P=0.024) and cardiorespiratory fitness (β=−0.019, P=0.003) predicting lower log HOMA-IRs. However, in our complete model (Model 4), including predicting and confounding variables (cardiovascular fitness, body fat percentage, age and sex), the association between cardiorespiratory fitness and log HOMA-IR became non-significant while MVPA remained significantly correlated with log HOMA-IR. When stratified by sex, our analysis did not show any evidence of moderation by sex on the association between MVPA and log HOMA-IR (data not shown).

**Table 2:** **Linear regression models predicting log of insulin resistance (log HOMA-IR**^**a**^**)**

**Beta (P-value)**	**Model 1**	**Model 2**	**Model 3**	**Model 4**
Physical Activity (30 min of MVPA^b^)	−0.2241	−0.1676	−0.0680	−0.1607
	(0.024)	(0.072)	(0.378)	(0.028)
Estimated VO2 Max^c^ (ml/kg/min)		−0.0144	0.0035	−0.0008
		(0.018)	(0.613)	(0.868)
Body Fat (%)			0.0396	0.0789
			(<0.001)	(<0.001)
Age (yrs.)				−0.0161
				(0.005)
Sex Male = 1; Female = 0				−1.054
				(<0.001)
Adjusted r2	0.042	0.064	0.165	0.423
r2 (% change)		52.4%	158%	156%

^a ^homeostatic model assessment of insulin resistance.

^b ^moderate-to-vigorous physical activity.

^c ^maximal oxygen consumption.

For all models n=402

### Light physical activity

We also examined minutes of light PA in our analysis. Although not statistically significant, there was a trend for an association between LPA and log HOMA-IR in our bivariate model (β=0.0288, P=0.099) and for LPA to predict log HOMA-IR when included in a more complete model with confounding variables (β=−0.0214, P=0.093). Importantly, when both LPA & MVPA were modeled with confounding variables, MVPA remained a significant predictor of log HOMA-IR (β=0.1457, P=0.035) while LPA (β=−0.0154, P=0.184) and cardiovascular fitness (β=0.0015, P=0.760) did not.

### Prediction of HOMA-IR based on Model 4

The predicted HOMA-IR means with confidence intervals for a range of MVPA levels are presented in Table 3. Assuming that the association found in Model 4 is causal, if the average individual in our model (based on means presented in Table 1 for predicting and confounding variables) who initially does not engage in any MVPA and subsequently increases his/her PA to 30 minutes of MVPA a day can decrease their HOMA-IR by 0.26 which is a 13% reduction in HOMA-IR.

**Table 3:** **Predicted effect of average minutes of MVPA**^**a **^**on HOMA-IR**^**b **^**and 95% confidence interval (95% CI)**

**Average MVPA (min/day)**	**HOMA-IR**	**95% CI**
0	1.95	1.58–2.32
15	1.81	1.57–2.06
30	1.69	1.52–1.86
45	1.57	1.40–1.73
60	1.46	1.25–1.69
75	1.35	1.08–1.63
90	1.26	0.93–1.59
105	1.17	0.79–1.55
120	1.09	0.67–1.51

Predictions are based on Model 4.

^a ^moderate-to-vigorous physical activity.

^b ^homeostatic model assessment of insulin resistance.

## Discussion

Although increased PA is known to improve IR, the mechanisms underlying this improvement are not completely understood. More specifically, it is difficult to distinguish the metabolic benefits of PA, per se, from the improvement in cardiorespiratory fitness that often accompanies an increase in PA. Presently, we found a strong and significant association between daily MVPA and a measure of IR (HOMA-IR) after adjusting for confounding variables including cardiorespiratory fitness. In contrast, we did not observe the same relationship between cardiorespiratory fitness and log HOMA-IR after correcting for the same confounding variables. Together these results highlight the potential importance of daily MVPA as a target intervention of improving IR independent of changes to cardiorespiratory fitness.

It is clear from previously published longitudinal studies that individuals with greater cardiorespiratory fitness are at lower risk for developing diabetes [18,19]. Yet improvements in cardiovascular fitness as a result of exercise training are highly variable and can take several months to develop [20]. Also, while the most robust improvements in cardiovascular fitness stem from higher intensity training [21,22], this kind of activity is also associated with greater participant discomfort and injury contributing to higher dropout rates [21]. Therefore, it is necessary to determine alternative intervention programs that may be more attainable by individuals at risk of developing diabetes. Our prediction analysis shows marked improvements in IR with relatively modest changes to daily MVPA. Indeed the benefits of cardiorespiratory fitness on other health outcomes such as cardiovascular health are undeniable [7,8], but modest PA may be an appropriate alternative for individuals that may see exercise intensity as a barrier to being more physically active.

Near-optimal fasting plasma glucose and insulin levels yield a HOMA-IR value close to 1, and our model showed that 120 minutes of MVPA per day predicts near optimal HOMA-IR values. This is equivalent to only 7.5 minutes of activity per hour during wakeful hours (accounting for 8 hours of sleep), throughout the day. Some PA guidelines emphasize a bout of at least 10 minutes of PA to improve health [23]. However, our analysis included accumulated minutes of PA throughout the entire day. We did not determine how the MVPA was accumulated, and future research is required to determine whether MVPA accumulated sporadically throughout the day is sufficient to improve IR or if it should be accumulated in bouts to have a positive effect on IR.

Surprisingly, LPA was not significantly associated with IR in our regression models. In contrast, similar methods, using objectively measured PA, identical criteria for defining intensity of PA, and inclusion of confounding variables like adiposity (i.e. waist circumference or BMI), have reported an inverse relationship between LPA and 2-hour plasma glucose concentrations during an oral glucose tolerance test [24]. This discrepancy between our results and previous analysis may be explained by the use of HOMA-IR, which has not been found to be associated with LPA [25]. Furthermore, while the use of accelerometers are currently the ideal method for measuring PA and have a high degree of sensitivity for LPA, HOMA-IR is a less sensitive measure of IR. Therefore LPA could have a positive effect on IR that we were unable to detect in our analysis. The use of more sensitive measures of IR (i.e. intravenous glucose tolerance test and hyperinsulinemic-euglycemic clamp methods) may be required to determine the influence of LPA on IR. Further investigation into the influence of LPA on IR may have important public health implications for individuals incapable or resistant to higher intensity PA.

Obesity is associated with increased risk of IR and type 2 diabetes [26,27]. In our complete model, we show that body fat percentage was most strongly associated with IR. With two-thirds of the U.S. population classified as overweight or obese, weight loss is an important therapeutic target for metabolic disease prevention [1,28]. In fact modest weight loss (~5% of initial body weight) can induce clinically relevant improvements in metabolic health [29]. However, clinical trials targeting behavioral changes to diet and exercise as well as pharmacological interventions have shown only modest weight loss and more importantly have proven difficult to sustain [30]. Because sustained weight loss is so difficult for many people [30], adopting a physically active lifestyle may be a more feasible alternative to weight loss for those at increased risk of developing IR.

Our study has certain limitations. Because our analysis is cross sectional, causation cannot be determined. However, our analysis showed that higher MVPA was indicative of lower IR in healthy adults. Large observational trials confirm the association between PA and HOMA-IR [31]. Controlled intervention trials in which adiposity, cardiorespiratory fitness and PA are manipulated independently in a sample large enough to convincingly prove causal association are likely to be infeasible. However, a more practical approach may be to systematically determine the minimum “dose” (i.e. intensity, duration, caloric expenditure, etc.) of PA, as well as the effect of habitual PA over time on the mechanisms underlying an improvement in IR.

It is also important to note that HOMA-IR is not the most sensitive measure of insulin resistance, and better measures of insulin resistance exist including an intravenous glucose tolerance test and hyperinsulinemic-euglycemic clamp methods. Although our analysis included a relatively insensitive measure of insulin resistance, we were able to detect measurable differences and the HOMA-IR method was ideal for measuring insulin resistance in a population this large and diverse.

The NHANES 2003–2004 survey limited cardiovascular fitness testing to individuals <49 yrs of age because of increased risk for an adverse cardiovascular incident. Examining the independent relationship between physical activity and cardiovascular fitness on insulin resistance in middle and older aged adults is an important question worth pursuing. However, we were unable to make this analysis with the current NHANES data set.

Because only healthy adults were selected for this analysis in order to rule out any unnecessary confounding factors brought about by disease, on average our sample was more active and fit than the average American. While participants averaged nearly 30 minutes of MVPA per day and had an estimated VO2max of 40 ± 9 ml·kg-1·min-1, there was a large range of values and high variability between participants, suggesting a rather heterogeneous subject pool rather than a highly fit population. Although a maximal exercise test was not performed, VO2max was estimated objectively using a submaximal exercise test. Submaximal exercise tests have been shown to be strongly correlated with maximal measures of VO2max [32]. Also, although the participants in the present study were generally younger than the age range where the incidence of newly diagnosed type 2 diabetes is greatest (45–64 years old [6]), in order to prevent or delay the onset of diabetes it is important to determine how PA behavior may influence IR in younger adults (>45 year old) in order to delay or prevent the onset. Other than requiring an overnight fast, diet and exercise were also not controlled prior to participants’ fasting blood draw or submaximal exercise testing. Although macronutrient meal composition and exercise can acutely alter IR [5,33] it is not unreasonable to assume that participant’s diets and exercise habits were not altered during this brief testing period.

## Conclusions

Our analysis, using objective measures of PA demonstrated that an increase in daily MVPA had a significant and positive effect on IR. Importantly these improvements were independent of other variables also known to influence diabetes risk including cardiovascular fitness and adiposity. Therefore, if improved glucose metabolism is the primary goal, lifestyle programs targeting improvements in metabolic health may be best designed to encourage individuals to participate in daily MVPA. This may be particularly important for individuals who have difficulty achieving or maintaining weight loss and/or for those who may be deterred by vigorous exercise regimens.

## Abbreviations

HOMA-IR: Homeostatic model of insulin resistance; MVPA: Moderate-to-vigorous physical activity; IR: Insulin resistance; PA: Physical activity; NHANES: National Health and Nutrition Examination Survey; VO2max: Maximal oxygen consumption; DEXA: Dual-energy X-ray absorptiometry.

## Competing interests

The authors declare that they have no competing interests.

## Authors’ contributions

RN, JH, RH, AS, SS, AK, CR conceived of the study, and participated in its design and coordination and helped to draft the manuscript. RN, RH and CR performed the statistical analysis. All authors read and approved the final manuscript.
